# Psychological Adaptation and Body Image in Women with Breast Cancer—The Role of Coping Strategies and Femininity

**DOI:** 10.3390/jcm15072640

**Published:** 2026-03-31

**Authors:** Marzanna Farnicka, Magdalena Kolańska-Stronka, Joanna Słowińska, Agata Poręba-Chabros

**Affiliations:** 1 Institute of Social Prevention and Resocialization, University of Warsaw, 00-721 Warsaw, Poland; 2 Institute of Psychology, SWPS University, 03-815 Warsaw, Poland; mkolanska@swps.edu.pl; 3 Wyższa Szkoła Nauk Pedagogicznych, 00-807 Warszawa, Poland; szostakasia@o2.pl; 4 Public Kindergarten nr 1, 63-300 Pleszew, Poland; 5 Institute of Psychology, John Paul II Catholic University of Lublin, 20-950 Lublin, Poland; agata.poreba@wp.pl

**Keywords:** breast cancer, body image, coping strategies, intimacy, illness acceptance, psychological adaptation, psycho-oncology

## Abstract

**Background:** Breast cancer poses not only a physical health threat but also significant emotional and identity challenges for women, particularly regarding femininity and body image. Understanding how patients adapt psychologically can guide effective psychosocial interventions. **Objective:** This study aimed to evaluate psychological adaptation, coping strategies, illness acceptance, and body image in women with breast cancer and identify factors associated with better adjustment. **Methods:** A cross-sectional study was conducted among 30 women aged 22–66 undergoing treatment at the Wielkopolskie Centrum Onkologii, Poland. Standardized tools included the Mini-MAC scale (coping strategies), Acceptance of Illness Scale (AIS), and Body Image Scale (BIS). Descriptive statistics and correlations were analyzed. **Results:** Most participants exhibited a constructive coping style, with positive redefinition and fighting spirit being predominant. Some women simultaneously showed elements of a destructive coping style, including helplessness and hopelessness, indicating complex emotional reactions. Overall, participants demonstrated high illness acceptance, despite notable body image-related discomfort, particularly shame, reduced perceived attractiveness, and appearance-related anxiety. While age did not correlate significantly with coping or body image, a significant negative association was found between age and illness acceptance, with younger women showing better adjustment. **Conclusions:** Psychological adaptation to breast cancer is multidimensional and individualized, dependent on personality traits, internal resources, and social support. Findings highlight the need for holistic, patient-centered psychosocial care, addressing both emotional adaptation and body image-related distress, including support for intimacy and prosthetic interventions. Individualized strategies can improve quality of life and functional outcomes during and after cancer treatment.

## 1. Introduction

Contemporary culture prioritizes youth, physical fitness, and success, leaving limited space for illness and its associated limitations. Cancer represents not only a threat to physical health but also a profound emotional and identity-related challenge, particularly for women when the disease affects domains closely linked to culturally constructed femininity [1]. Fear of death, changes in daily functioning, and treatment-related alterations in appearance generate significant psychological distress, which may lead either to crisis or to identity redefinition and mobilization of personal resources.

Femininity is a multidimensional construct encompassing psychological traits, social roles, and physical attributes shaped by cultural and social norms [2,3]. Stereotypically, it is associated with emotionality, empathy, caregiving, and physical delicacy. Female identity and gender-related values influence interpersonal functioning across family, intimate, social, and professional domains, integrating biological characteristics with culturally embedded expectations that shape self-perception and social evaluation [4]. In this work, the term ‘femininity’ is employed to refer to culturally prescribed traits and expectations associated with being female, whereas ‘female identity’ is used to denote the process of developing and understanding one’s own sense of self as a woman. The authors maintain this distinction because ‘female identity’ captures the individual, psychological process of constructing one’s sense of self as a woman, whereas ‘femininity’ reflects the sociocultural dimensions that influence how women cope with illness.

Motherhood remains a significant dimension of femininity and female identity and can be understood as one of its biological characteristics, as it is rooted in reproductive capacity. At the same time, it encompasses important psychological and social components, including emotional bonding and caregiving roles. While motherhood allows for the realization of the biological aspects of womanhood, femininity itself should not be defined solely through this lens [5]. Moreover, physical and social changes related to pregnancy and childcare may both strengthen and challenge self-acceptance. Sexuality constitutes another essential dimension of femininity, relevant to both social functioning and intimate relationships.

Cancer may substantially disrupt the physical and psychological dimensions of female well-being, affecting body image, self-esteem, and identity. Treatment-related changes in appearance often conflict with culturally promoted ideals of the female body, despite increasingly inclusive contemporary definitions of femininity [6]. Empirical evidence indicates that negative body image is associated with poorer quality of life and psychological well-being among women with cancer [7,8]. Conversely, maintaining attention to appearance—even in a limited form—may regulate emotions and restore a sense of agency and normality during treatment. Although appearance satisfaction does not affect treatment efficacy, it significantly influences mental well-being and quality of life, indirectly supporting recovery processes [9].

The experience of cancer often leads to a redefinition of female identity, shifting the focus from physical appearance to psychological resilience, emotional strength, and acceptance of personal limitations [10]. Psychological attributes commonly associated with femininity, such as adaptability, empathy, and interpersonal competence, may function as key resources facilitating coping with illness and adjustment to altered life circumstances [2].

Breast cancer is the most frequently diagnosed malignant neoplasm among women worldwide and remains one of the leading causes of cancer-related mortality. Approximately 1.7 million new cases are diagnosed annually, with over 500,000 deaths attributed to the disease. Incidence rates are highest in industrialized countries and lowest in Africa and Southeast Asia [11,12]. In Poland, breast cancer represents a major public health concern, with most cases diagnosed after the age of 50. Although rare, male breast cancer accounts for approximately 1% of cases [13]. Young women diagnosed before the age of 40 constitute a distinct clinical group, often experiencing a more aggressive disease course and additional psychosocial challenges, including fertility-related concerns. Despite representing a smaller proportion of patients, incidence in this age group is increasing globally [12].

A cancer diagnosis constitutes a profound emotional crisis requiring psychological adaptation. Social perceptions of cancer are often shaped by negative stereotypes associating the disease with death, suffering, and helplessness, which may contribute to stigmatization and social withdrawal [1,14]. Persistent myths portraying cancer as invariably fatal or as a consequence of personal lifestyle choices may lead to patient blaming and social condemnation. Women with breast cancer are particularly vulnerable to social pressure related to perceived loss of attractiveness and femininity, which may adversely affect intimate relationships and social functioning [1,15].

Adaptation to cancer typically follows a stage-based process, including shock and denial, anger, anxiety and depression, and eventual adjustment and acceptance. Coping strategies play a crucial role in this process. Constructive coping styles—such as fighting spirit and positive reappraisal—are associated with better psychological functioning and quality of life, whereas destructive styles—characterized by helplessness, hopelessness, and anxious preoccupation—are linked to poorer outcomes [16,17].

Higher levels of self-efficacy enhance treatment adherence, engagement in health-promoting behaviors, and the use of active coping strategies [18]. In women with breast cancer, constructive coping is associated with better emotional, cognitive, and social functioning, as well as a greater ability to maintain professional and family roles. In some cases, the cancer experience may also lead to positive psychological changes, referred to as benefit finding, including re-evaluation of life priorities, strengthened relationships, and personal growth [19]. Studies have shown the importance of a good social environment in that process [20].

Breast cancer presents not only a medical but also a psychological and social challenge. Effective treatment and quality of life depend on comprehensive, multidisciplinary care that integrates medical, psychological, and rehabilitative support [7]. Psychological adaptation to cancer is a dynamic process influenced by multiple factors, including diagnosis, treatment course, physical well-being, family situation, financial status, and social support [21,22]. The prospect of a life-threatening illness with prolonged treatment and uncertain outcomes can intensify anxiety, helplessness, and emotional distress. At the same time, cancer may also activate psychological resources, fostering resilience, a fighting spirit, and acceptance of illness.

The present study aimed to evaluate psychological adaptation in women with breast cancer, with particular emphasis on coping strategies, illness acceptance, and personal discomfort connected with body image [20]. The main research question was formulated as follows: How do women with breast cancer psychologically adapt to the disease and treatment, and which factors facilitate better adaptation?

It was hypothesized that higher illness acceptance and constructive coping styles are associated with better psychological adaptation and lower perceived discomfort, whereas destructive coping styles are linked to greater discomfort. Although coping strategies, illness acceptance, and body image have often been studied as related aspects of psychological adaptation in cancer patients, emerging evidence suggests that these domains may operate independently. Recognizing their potential dissociation allows for a more nuanced understanding of adjustment, as a patient may accept her illness without necessarily experiencing reduced body image distress or may employ effective coping strategies while struggling with self-perception. Therefore, the present study adopts a conceptual framework that treats these domains as potentially distinct processes, enabling a more precise examination of the factors contributing to psychological adaptation in women with breast cancer.

Additionally, age was assumed to mediate the relationship between body image and illness acceptance.

The age cutoff of 35 was chosen based on both epidemiological and developmental considerations. Epidemiologically, 35 years is often used to distinguish younger women with breast cancer, who may experience different disease courses and have greater fertility-related concerns [23]. From a developmental perspective, 35 marks the end of early adulthood [24,25], a period when women are more likely to be planning or considering motherhood [26]. However, due to the lack of significant differences between the younger and older groups in our sample, age was ultimately treated as a continuous variable in the analyses, allowing for a more precise examination of its relationship with psychological adaptation and body image.

## 2. Materials and Methods

### 2.1. Participants and Procedure

The study was conducted using a questionnaire-based design in a sample of 30 women diagnosed with breast cancer who were treated at the Greater Poland Cancer Centre (Wielkopolskie Centrum Onkologii, WCO) in Poznań. Data were collected between February and April 2025. A total of 60 patients were invited to participate in the study; subsequently, 15 patients resigned, and 15 questionnaires were excluded due to being filled out improperly. At the time of the study, all participants were undergoing active oncological treatment. All patients gave written informed consent to participate in the study, to complete the questionnaires, and to publish their information. This research was conducted in accordance with the Declaration of Helsinki and the Ethical Principles of Psychologists and Code of Conduct. The Commission of Research at the Higher School of Pedagogical Sciences in Warsaw approved the research. All personally identifiable information, such as names and identification numbers, was removed from the dataset and replaced with unique coded identifiers to maintain confidentiality and strictly protect the anonymity of the medical records throughout the study.

### 2.2. Measures

Three standardized instruments were used in the study.

Psychological adaptation to cancer was assessed using the Mini-Mental Adjustment to Cancer (Mini-MAC) scale developed by Greer, Moore, and Watson [27,28], in its Polish adaptation [29]. The questionnaire consists of 29 statements evaluating four strategies for coping with the disease:

− Anxious preoccupation: Reflects anxiety and the perception of the illness as an uncontrollable threat.
− Fighting spirit: Motivates the patient to take active steps to overcome the illness, viewed as a personal challenge.
− Helplessness–hopelessness: Indicates a sense of impotence and giving in to the illness.
− Positive redefinition: Involves re-evaluating the situation to find hope while remaining aware of the illness’s gravity.

These strategies are grouped into two broader coping styles: constructive (fighting spirit, positive redefinition) and destructive (anxious preoccupation, helplessness–hopelessness). Responses are rated on a 4-point Likert scale (1–4). Scores for each subscale range from 7 to 28, where higher scores indicate a higher intensity of the given strategy. Clinical interpretation is based on sten scores. The tool demonstrates satisfactory reliability, with Cronbach’s alpha coefficients above 0.70.

The Acceptance of Illness Scale (AIS), developed by Felton et al. [30] and adapted into Polish by Juczyński [29,31], was used to measure illness acceptance. The scale contains eight items addressing the negative consequences of illness, such as physical limitations, dependence on others, and reduced self-esteem. Responses are provided on a 5-point scale (1–5). The total score ranges from 8 to 40; higher scores (e.g., 8–10 sten) indicate greater illness acceptance, lower emotional distress, and better psychological adjustment. The AIS is particularly suitable for oncology patients, as it helps in planning targeted psychotherapeutic interventions.

The Body Image Scale (BIS), developed by Hopwood et al. [32] and adapted into Polish [29,33], was used to assess body image. This 10-item, one-dimensional self-report instrument evaluates emotional, cognitive, and social aspects of body image, including shame, reduced perceived attractiveness, and appearance-related anxiety. Each item is rated on a 4-point Likert scale (0–3). Total scores range from 0 to 30, where higher scores indicate greater psychological discomfort. A score of ≥ 10 points is typically interpreted as a clinically significant body image disturbance, often requiring psycho-oncological intervention. The scale shows high reliability (Cronbach’s alpha = 0.93) and good clinical validity.

### 2.3. Statistical Analysis

Statistical analyses were performed using IBM SPSS Statistics version 28.0. Descriptive statistics (means, standard deviations, medians, and ranges) were calculated for all variables. Distribution normality was assessed using the Shapiro–Wilk test; all variables approximated normal distributions (all *p* > 0.05). Bivariate relationships were examined using Pearson product–moment correlation coefficients. To enhance the reliability of the estimates and account for the small sample size, the bootstrap method was employed (simple sampling, 1000 resamples). Results are reported with 95% percentile confidence intervals (95% CIs). Age group comparisons were performed using the non-parametric Mann–Whitney U test, with statistical significance set at α = 0.05 (two-tailed). Effect sizes (r) were calculated as Z/√N and interpreted according to Cohen’s (1988) [34] conventions: small (0.10–0.29), medium (0.30–0.49), and large (≥0.50).

Artificial intelligence (AI) tools were used exclusively to assist with language translation and editorial refinement of the manuscript. The AI support was limited to improving grammar, clarity, and linguistic accuracy. The authors take full responsibility for the content and integrity of the manuscript.

## 3. Results

### 3.1. Descriptive Analyses

The study group consisted of 30 women. The age was from 22 to 66 years (*M* = 45.5, *SD* = 13.8, *Mdn* = 45.5). The sample was divided into two age groups for comparative analyses: younger women (≤35 years, *n* = 8, 26.7%) and older women (>35 years, *n* = 22, 73.3%).

Constructive coping predominated among the participants (*M* = 46.77; *SD* = 2.11), with positive reappraisal showing the highest mean scores. Destructive coping was reported at a moderate level (*M* = 17.93; *SD* = 2.88). Detailed descriptive statistics for all psychological variables are presented in Table 1.

Participants reported high levels of illness acceptance (*M* = 34.67; *Mdn* = 35.00; *SD* = 2.96). Most women adapted well to health limitations. Specifically, activity restrictions scored moderately (*M* = 4.17), while dependence on others scored the lowest (*M* = 3.03), and feelings of being unnecessary were moderate (*M* = 3.67). Most participants did not perceive themselves as a burden or experience significant social stigma.

Body image discomfort was moderate-to-high (*M* = 17.20; *SD* = 1.99). The most frequently reported difficulties included reduced perceived attractiveness, diminished sense of femininity, and discomfort in intimate situations. Conversely, social avoidance and intense shame were rarely reported.

### 3.2. Relationships Among Variables

*Within-Scale Relationships*: Within the destructive coping style, a significant moderate positive correlation was found between helplessness–hopelessness and anxious preoccupation (*r* = 0.61, *p* < 0.001, 95% CI [0.31, 0.79]). This confirms that women who feel helpless also tend to experience heightened anxiety.

In contrast, within constructive coping, fighting spirit and positive redefinition showed a strong positive trend, although the result did not reach the conventional threshold for statistical significance (*r* = 0.36, *p* = 0.053, 95% CI [−0.00, 0.63]). This suggests that while active, challenge-oriented approaches and existential reframing tend to coexist, they may function as relatively distinct adaptive processes in this patient group. Given the proximity to significance and the bootstrap confidence interval, this relationship warrants further investigation in a larger cohort.

*Inter-Scale Correlations*: Pearson correlation analyses, supplemented with bootstrap-estimated 95% confidence intervals, did not reveal significant associations between overall coping styles, illness acceptance, and body image (Table 2). Specifically, body image discomfort was not significantly correlated with either constructive coping (*r* = 0.06, *p* = 0.750, 95% CI [−0.31, 0.41]) or destructive coping (*r* = −0.26, *p* = 0.161, 95% CI [−0.57, 0.11]), suggesting that body image may function as an independent domain of psychological adjustment in this sample.

Similarly, constructive coping was not significantly associated with illness acceptance (*r* = 0.28, *p* = 0.135, 95% CI [−0.09, 0.58]), although the effect size was small-to-moderate. The association between constructive and destructive coping was also non-significant (*r* = −0.27, *p* = 0.150, 95% CI [−0.57, 0.10]).

In contrast, a statistically significant and strong negative correlation was observed between destructive coping and illness acceptance (*r* = −0.61, *p* < 0.001, 95% CI [−0.79, −0.31]), indicating that lower levels of illness acceptance are associated with a greater tendency toward maladaptive coping strategies. This effect can be interpreted as large according to Cohen’s conventions.

Regarding age, a statistically significant moderate negative correlation was found with illness acceptance (*r* = −0.44, *p* = 0.015, 95% CI [−0.69, −0.09]), suggesting that older participants reported lower levels of illness acceptance. The correlation between age and constructive coping approached statistical significance (*r* = 0.36, *p* = 0.053, 95% CI [−0.00, 0.64]), indicating a potential trend toward higher constructive coping in older women, although this finding should be interpreted with caution. No significant associations were found between age and destructive coping (*r* = 0.19, *p* = 0.311) or body image (*r* = 0.10, *p* = 0.585).

### 3.3. Age Group Comparisons

Differences between younger (≤35 years, *n* = 8) and older (>35 years, *n* = 22) women were examined using the Mann–Whitney U test (see Table 3). Effect sizes were calculated as *r* = *Z*/√*N*.

No statistically significant differences were found for constructive coping, *U* = 121.50, *Z* = 1.62, *p* = 0.118, *r* = 0.30, or destructive coping, *U* = 96.00, *Z* = 0.39, *p* = 0.730, *r* = 0.07. The difference in body image approached statistical significance, *U* = 129.00, *Z* = 1.95, *p* = 0.056, *r* = 0.36, indicating a moderate effect size despite the lack of statistical significance.

In contrast, a statistically significant difference was observed for illness acceptance, *U* = 32.50, *Z* = −2.64, *p* = 0.007, *r* = 0.48, representing a moderate-to-large effect. Younger women reported higher levels of illness acceptance compared to older women.

Overall, although most group differences were not statistically significant, several effects (constructive coping and body image) reached small-to-moderate or moderate magnitude, suggesting potentially meaningful trends that may not have reached significance due to the small sample size.

## 4. Discussion

The findings of the present study provide a nuanced perspective on the psychological functioning of women undergoing treatment for breast cancer, highlighting the complexity and multidimensional nature of adjustment processes in a contemporary cultural context that emphasizes physical appearance and social roles [1,2]. A central observation is the predominance of constructive coping strategies, such as fighting spirit and positive redefinition, alongside high levels of illness acceptance.

The initial hypothesis, assuming that higher illness acceptance and constructive coping styles are associated with better psychological adaptation, was partially supported. A significant, strong negative correlation was found between destructive coping and illness acceptance. This confirms that helplessness–hopelessness and anxious preoccupation are major barriers to psychological adjustment, echoing the clinical observations [29,35]. In the Polish clinical context, a lack of illness acceptance often manifests as a sense of being a burden, which reinforces destructive cognitive patterns [31].

In contrast, the relationship between constructive coping and illness acceptance did not reach statistical significance. While meta-analytic evidence [16] generally associates active coping with better well-being, our results must be interpreted with caution due to the small sample size. The limited statistical power increases the risk of Type II error, potentially obscuring a medium-sized effect. Therefore, this result should be viewed as inconclusive rather than definitive proof of independence between these constructs.

One of the most striking findings is the dissociation between illness acceptance and body image discomfort. The hypothesis that higher illness acceptance would mitigate body image distress was not supported. Participants demonstrated high illness acceptance while simultaneously reporting clinically significant body image disturbance. This suggests that accepting the “patient role” and functional limitations is a distinct process from accepting the “altered self” in the context of femininity and attractiveness. Polish women’s identity is deeply rooted in cultural constructions of femininity that integrate biological attributes with social expectations [2,3].

At this point, it is important to note that the study focused on two distinct domains of psychological adaptation: illness acceptance and body image. The Acceptance of Illness Scale (AIS) [29,30,31] was used to measure illness acceptance, focusing on autonomy, self-worth, and the negative consequences of disease, such as physical limitations and dependence on others. Higher AIS scores indicate greater acceptance and better psychological adjustment. The Body Image Scale (BIS) [29,32,33] assesses body-related distress after cancer treatment, including shame, reduced perceived attractiveness, and appearance-related anxiety, with higher scores reflecting greater discomfort. Importantly, these scales measure distinct constructs: a patient may accept her illness cognitively while still experiencing significant distress regarding her body. Recognizing these domains as potentially independent allows for a more nuanced understanding of psychological adaptation in women with breast cancer. This dissociation highlights that “general” adaptation does not automatically resolve identity-related concerns, requiring a separate trajectory of psychological restructuring [15,33].

The study utilized a cutoff of 35 years to distinguish “younger” women from early adulthood [22,23], reflecting the distinct challenges of this group, such as fertility-related concerns and more aggressive disease courses [11,12]. The hypothesis regarding age as a mediator was partially challenged. Younger women (≤ 35) showed significantly higher illness acceptance than older women. This may reflect a more intensive mobilization of personal resources or “benefit finding” [19] in the face of a life-disrupting event at an early age. Alternative explanations of this finding may suggest that older women experience greater difficulty coping with this dimension because they perceive the negative consequences of illness more acutely, including physical limitations, dependence on others, and diminished self-esteem. This heightened vulnerability may stem from cumulative life burdens, lower dispositional optimism, or reduced vitality. Importantly, these factors may themselves be shaped by sociocultural conditioning, including culturally prescribed gender roles and expectations regarding aging and femininity.

However, age did not significantly correlate with body image distress or coping styles. This suggests that the psychological burden of cancer transcends age groups, consistent with recent findings [36,37].

This finding underscores the critical role of cultural and sociocultural determinants in shaping psychological outcomes. In Poland, threats to femininity and identity constitute a persistent source of distress across the lifespan. Concerns related to body image and coping are influenced not only by individual and developmental factors (e.g., age) but also by culturally embedded ideals of femininity [6] and structural characteristics of the healthcare system, including low confidence in treatment [38] and not enough supportive environment, such as nursing services or social care [20,21]. In a sociocultural context where femininity is closely linked to physical attractiveness [1,3,4], these factors may exert a particularly strong impact on older women. However, it remains unclear whether the observed patterns reflect culture-specific dynamics or a more universal phenomenon. Although globalization and widespread internet access may contribute to the diffusion of similar norms across contexts, further cross-cultural research is necessary to disentangle these effects.

The study partially confirmed the hypotheses presented in the Introduction. The relationship between constructive coping and illness acceptance did not reach statistical significance, indicating partial support for this hypothesis. Destructive coping strategies significantly increased perceived discomfort and hindered psychological adaptation, confirming the corresponding hypothesis. The hypothesis regarding positive body image was not supported—participants could accept their illness while simultaneously experiencing significant body image distress. Age partially influenced illness acceptance, with younger women showing higher acceptance, but it was not significantly related to body image distress or coping strategies, indicating a limited mediating role. These findings are part of a broader highlight of the complexity of psychological adaptation in women with breast cancer [39] and underscore the need to address body image and individual coping strategies separately from general illness acceptance.

## 5. Limitations and Future Research

Several limitations of this study must be acknowledged. First, the small sample size (*N* = 30) recruited from a single oncology center restricts the generalizability of the findings and results in low statistical power. This increases the risk of a Type II error, meaning that non-significant correlations—such as those between coping styles and illness acceptance—should be viewed as inconclusive rather than definitive proof of independence. Second, the dichotomization of age at 35 years, while clinically justified, may have reduced statistical variance; future research should treat age as a continuous variable in larger cohorts to capture more nuanced trends. Third, the study relied on self-report questionnaires, which may be subject to social desirability bias or subjective response errors.

The cross-sectional design captures psychological adaptation at a single point in time, thereby failing to account for the dynamic changes that occur over the full course of treatment and long-term recovery. Finally, other significant factors that may influence adaptation—such as socioeconomic status, pre-existing mental health conditions, or the specific quality of social support networks—were not extensively analyzed.

The finding highlights the importance of considering cultural and sociocultural factors. Based on these results, it cannot be assumed that body image concerns and coping challenges represent a universal trend; they appear to be sociocultural context-specific. Although globalization and internet access suggest that similar patterns might occur in other cultural contexts, this area clearly warrants further research.

Future research should address these limitations by employing larger, longitudinal, and multi-center designs. Future research should also consider cultural and sociocultural factors shaping women’s experiences of femininity and coping with illness, including key indicators of femininity such as physical attractiveness, motherhood, and socially prescribed roles. It is important to examine how the development of female identity interacts with evolving societal standards of female roles and appearance, particularly among older or ill women. Additional factors to consider include the level of medical care and trust in healthcare providers, age-related stereotypes, time since diagnosis, and the presence or absence of children, as these may influence self-perception, coping strategies, and psychological adaptation. Integrating these elements can provide a more comprehensive understanding of how individual identity and cultural norms jointly shape women’s adjustment to illness.

Such approaches would provide a more comprehensive understanding of the complex dynamics of psychological adaptation and the long-term trajectory of illness acceptance in women with breast cancer.

## 6. Conclusions

Psychological adaptation to cancer is a multidimensional and dynamic process, influenced by personality traits, internal resources, social support, and socioeconomic context. The results of this study suggest that women undergoing or recovering from cancer treatment may benefit from individually tailored psychological support and comprehensive psychosocial rehabilitation programs.

This analysis revealed three primary findings: (1) Independence of adaptation dimensions: Coping strategies, illness acceptance, and body image appear to function as largely independent processes rather than a single unified construct. (2) Dissociation between acceptance and body image: Participants demonstrated that high illness acceptance can coexist with significant body image distress, indicating that general adaptation does not automatically mitigate identity- or appearance-related concerns. (3) Age-related trends: Age was significantly associated only with illness acceptance, with younger women showing higher acceptance. No significant correlations were found between age and destructive coping or body image, while a trend toward higher constructive coping in older women was observed but did not reach statistical significance.

The independence of these adaptive dimensions suggests that clinical practice must move toward domain-specific interventions. Key recommendations based on the findings and latest literature [7,8,16] include the following:

(1) **Targeted Body Image Support:** Ensuring access to prosthetic counseling and psychological strategies to manage changes in appearance, as appearance satisfaction indirectly supports recovery [9].
(2) **Intimate Health Guidance:** Providing specific support for maintaining intimate relationships, as sexual function is a critical but often neglected dimension of well-being [15].
(3) **Holistic Nursing Care:** Oncology nurses should play a pivotal role as educators and motivational support, addressing not just procedural needs but also the patient’s psychological comfort [25,26].
(4) **Support Groups as “Social Mirrors”:** Utilizing group settings to facilitate emotional insight and restore a sense of agency, helping women navigate cultural pressures regarding female appearance [7,14].
(5) **Addressing Changing Sociocultural Expectations:** Recognizing that societal standards and expectations regarding appearance, sexuality, and roles often shift for older or ill women, interventions should support the development of female identity while helping women navigate evolving notions of femininity.

The present study underscores the importance of considering cultural and sociocultural factors, as body image concerns and coping challenges are context-specific rather than universal. While globalization and internet access may produce similar patterns elsewhere, further research is needed. Future studies should integrate interventions that support the development of female identity while helping women navigate evolving societal standards of femininity, particularly among older or ill women. Approaches such as targeted body image support, intimate health guidance, holistic nursing care, and support groups as “social mirrors” may enhance coping and psychological adaptation in ways that are both personally meaningful and culturally sensitive, providing a comprehensive understanding of how sociocultural norms shape individual adjustment to illness.

Ultimately, fostering insight into these adaptive processes can empower patients to realign their lives with current needs, promoting resilience, recovery, and a sustained sense of well-being after a cancer diagnosis.

## Figures and Tables

**Table 1:** Descriptive statistics for all study variables (*N* = 30).

Variable	Range	Mean (M)	SD	Median	Sten	Notes
Constructive coping	14–56	46.77	2.11	48	7–8	Combined subscales
Destructive coping	14–56	17.93	2.88	18	1	Combined subscales
Illness acceptance (AIS)	8–40	34.67	2.96	35	8–9	Higher = better acceptance
Body image (BIS)	0–30	17.20	1.90	17	>10 *	Higher = greater discomfort

* Score ≥ 10 indicates clinically significant disturbance.

**Table 2:** Intercorrelations among study variables.

Variable	1	2	3	4
1. Constructive coping	—			
2. Destructive coping	−0.27 [−0.57, 0.10]	—		
3. Illness acceptance	0.28 [−0.09, 0.58]	−0.61 ** [−0.79, −0.31]	—	
4. Body Image	0.06 [−0.31, 0.41]	−0.26 [−0.57, 0.11]	−0.03 [−0.39, 0.34]	—
5. Age	0.36 [−0.00, 0.64]	0.19 [−0.18, 0.52]	−0.44 * [−0.69, −0.09]	0.10 [−0.27, 0.45]

* Note: Values are Pearson’s *r* coefficients; 95% confidence intervals (in brackets) were estimated using bootstrap (1000 resamples) and confirmed using Fisher’s z-transformation. * *p* < 0.05, ** *p* < 0.01. Effect size interpretation (Cohen, 1988 [34]): small (|r| = 0.10–0.29), medium (|r| = 0.30–0.49), large (|r| ≥ 0.50).

**Table 3:** Comparison of psychological variables by age group.

Variable	Younger (≤35) *M* (*SD*)	Older (>35) *M* (*SD*)	*U*	*Z*	*p*	Effect Size
Constructive coping	45.50 (2.88)	47.23 (1.60)	121.50	1.62	0.118	0.30
Destructive coping	17.50 (1.85)	18.09 (3.19)	96.00	0.39	0.730	0.07
Illness acceptance	36.38 (3.70)	34.05 (2.46)	32.50	−2.64	0.007 *	0.48
Body Image	16.00 (1.51)	17.64 (1.99)	129.00	1.95	0.056	0.36

Note: Values are presented as mean (*SD*). Mann–Whitney *U* tests were used for group comparisons. Effect size r was calculated as *Z*/√*N* and interpreted as small (0.10–0.29), medium (0.30–0.49), and large (≥0.50). * *p* < 0.05.

## Data Availability

The data presented in this study are available upon request from the corresponding author. The data are not publicly available due to privacy and ethical restrictions.

## Abbreviations

The following abbreviations are used in this manuscript:

WCO	Greater Poland Cancer Centre (Wielkopolskie Centrum Onkologii)
AIS	Acceptance of Illness Scale
BIS	Body Image Scale
Mini-MAC	Mini-Mental Adjustment to Cancer
M	Mean
SD	Standard deviation
Mdn	Median
CV	Coefficient of Variation
CI	Confidence interval
